# Supplementary material for: A Therapeutic Pathway in Patients with Chronic Coronary Syndromes: Proposal for Optimization
Source: J Clin Med. 2022 Apr 8;11(8):2091. doi: 10.3390/jcm11082091 (PMC9028169; doi:10.3390/jcm11082091)
Supplement: Supplementary file 1 [file jcm-11-02091-s001.zip › Supplementary Material 1-Questionnaires.pdf]

Supplementary Material 1 - Questionnaires

QUESTIONNAIRE 1

SECTION Respondent Profile

a. First Name/Surname: ....
b. Year of Birth: ....
c. Year of Graduation: ....
d. Specialization: ....
e. Facility where you work: ....
f. Prevailing Scope of Practice:
   ❍ Hemodynamics Room
   ❍ Cardiac Intensive Care Unit / Department
   ❍ Outpatient clinic
g. How many years have you been involved in CV diseases: ....
h. Your role in the facility: ....
i. Are you a member of one or more scientific societies?
   ❍ Yes
   ❍ No

If yes, which ones? ....

SECTION The Current Framework

1. **You work in a facility:**
   [multiple answers can be selected]
   a. ☐ University
   b. ☐ Public hospital
   c. ☐ Private hospital
   d. ☐ Hub Hospital
   e. ☐ Spoke Hospital
   f. ☐ Territorial cardiology service (outpatient clinic)

2. **What is the level of technology implementation in your facility?**
   [Please express the level of implementation for each answer choice by selecting a score between 1 (lowest grade) and 9 (highest grade)]

a.	Computerized medical records	1 2 3 4 5 6 7 8 9
b.	Computerized discharge letter	1 2 3 4 5 6 7 8 9
c.	Automated follow-up visit	1 2 3 4 5 6 7 8 9
d.	Prescription management software	1 2 3 4 5 6 7 8 9

3. **To manage the follow-up of CCS patients does your facility use alternative remote modalities?**
   a. ❍ Yes
   b. ❍ No and there is no project in progress
   c. ❍ No, but there is a project underway

4. **To whom are patients in periodic follow-up with CCS that you see at your facility referred?**
   [multiple answers can be selected]

a. ☐ General Practitioner
b. ☐ Diabetologist or other specialist
c. ☐ Emergency room

5. **A patient with CCS post-MI is placed on ticagrelor 60 mg therapy:**
[multiple answers can be selected]
a. ☐ By my facility and followed structurally with a dedicated post-acute pathway
b. ☐ By my facility even if there is no dedicated post-acute pathway
c. ☐ Not by my facility but by another facility of my Hospital and there is a dedicated pathway
d. ☐ Not by my facility but by another facility of my Hospital and there is no dedicated pathway
e. ☐ In my network every cardiologist is qualified to prescribe ticagrelor 60 mg and at one year can decide to start the treatment independently.

6. **Who in your experience suggests that DAPT should be extended?**
[multiple answers can be selected]
a. ☐ Interventional cardiologist
b. ☐ Clinical cardiologist at discharge
c. ☐ Clinical cardiologist at outpatient follow-up
d. ☐ Territorial cardiologist

7. **Do you use SCOREs to define ischemic and/or hemorrhagic risk?**
a. ❍ Yes
b. ❍ No
If yes, which ones ....

8. **A patient with ACS who comes to your observation is:**
a. ❍ Inserted in a follow-up pathway at the structure
b. ❍ Referred to an outpatient cardiologist who takes care of him/her
c. ❍ Informed about a general follow-up and the patient chooses where to be followed.

9. **In your facility, regarding pretreatment with P2Y12 inhibitor for the treatment of the patient with STEMI, which of the following statements corresponds to your reality?**
[multiple answers can be selected]
a. ☐ Pretreatment with inhibitor is performed in ambulance
b. ☐ Pretreatment is performed in the Spoke center
c. ☐ Pretreatment is performed in the Hub center
d. ☐ Pretreatment is not performed

10. **In your facility, for the treatment of the patient with NSTE-ACS, which of the following statements correspond to your reality?**
[multiple answers can be selected]
a. ☐ In case of coronarography within 24 hours, pretreatment with P2Y12 inhibitor is not performed
b. ☐ Pretreatment is also performed in many cases of patients receiving coronarography within 24 hours
c. ☐ Pretreatment is performed in cases of coronarography beyond 24 hours
d. ☐ Pretreatment is never performed

11.a In your facility, for the patient with STEMI who requires P2Y12 inhibitor you preferentially choose:

[order by importance the response options indicating with 1 the preferred or most frequent choice and with 3 the least frequent choice]

...... Clopidogrel
...... Ticagrelor
...... Prasugrel

11.b. The choices in 11.a are made preferentially by:

a. ❍ Ambulance physician
b. ❍ Emergency Room Physician
c. ❍ Interventional cardiologist
d. ❍ Clinical cardiologist

12.a In your facility, for the patient with NSTE-ACS who requires P2Y12 inhibitor, you choose preferentially:

[order by importance the response options indicating with 1 the preferred or most frequent choice and with 3 the least frequent choice]

...... Clopidogrel
...... Ticagrelor
...... Prasugrel

12.b. The choices in 12.a are made preferentially by:

a. ❍ Ambulance physician
b. ❍ Emergency Room Physician
c. ❍ Interventional cardiologist
d. ❍ Clinical cardiologist

13. Is there a hospital protocol in the choice of P2Y12 inhibitor?

a. ❍ Yes
b. ❍ No
c. ❍ Not applicable if respondent is an outpatient physician

14. In your facility, is the indication to continue dual antithrombotic therapy beyond 12 months suggested in the discharge letter when appropriate?

a. ❍ Yes
b. ❍ No
c. ❍ Not applicable if respondent is an outpatient physician

15. If you work mainly in an outpatient setting, how important do you think it is that at discharge you are advised whether or not to continue DAPT beyond 12 months?

[Please indicate the importance you attribute to this indication by selecting a score between 1 (minimum importance) and 9 (maximum importance)] 1 2 3 4 5 6 7 8 9

16. If you work mainly in an outpatient clinic, which elements hinder your choice to continue DAPT beyond 12 months?

[Please indicate the importance you attach to each answer choice by selecting a score between 1 (least importance / least obstacle) and 9 (greatest importance / greatest obstacle)]

a. Low knowledge of the PCI intervention 1 2 3 4 5 6 7 8 9
b. Poor knowledge of the patient's ischemic risk profile 1 2 3 4 5 6 7 8 9

c. Fear of bleeding risk 1 2 3 4 5 6 7 8 9
d. Limited margin of benefit 1 2 3 4 5 6 7 8 9
e. Other (specify) 1 2 3 4 5 6 7 8 9

SECTION The Expert Opinion

17. How would you define a patient with CCS?
[Express your level of agreement for each response option by selecting a score between 1 (maximum disagreement) and 9 (maximum agreement)]
a. Patient with suspected CAD and stable angina and/or dyspnea 1 2 3 4 5 6 7 8 9
b. Symptomatic patient 1 2 3 4 5 6 7 8 9
c. Asymptomatic patient (and/or with stabilized symptoms) 1 2 3 4 5 6 7 8 9
d. Patient who has had the acute event 1 2 3 4 5 6 7 8 9
e. Patient with elective revascularization for more than one year 1 2 3 4 5 6 7 8 9
f. Patient more than one year after MI with no additional events 1 2 3 4 5 6 7 8 9
g. Patient with elective revascularization for less than one year 1 2 3 4 5 6 7 8 9
h. Patient with microvascular or vasospastic angina 1 2 3 4 5 6 7 8 9
i. Patient with recent episode of heart failure and/or diagnosis of left ventricular dysfunction 1 2 3 4 5 6 7 8 9

18. Do you consider CCS a dynamic disease?
[Please express your level of agreement with this statement by selecting a score between 1 (maximum disagreement) and 9 (maximum agreement)] 1 2 3 4 5 6 7 8 9

19. How important is it for a patient with post-MI CCS and multiple risk factors to have the multidisciplinary collaboration of:
[Please express your level of agreement for each response option by selecting a score between 1 (maximum disagreement) and 9 (maximum agreement)]
a. Diabetologist 1 2 3 4 5 6 7 8 9
b. Nutritionist / Dietitian 1 2 3 4 5 6 7 8 9
c. Psychologist 1 2 3 4 5 6 7 8 9
d. Physiotherapist 1 2 3 4 5 6 7 8 9
e. Nephrologist 1 2 3 4 5 6 7 8 9

20. For an ACS patient treated with medical therapy alone, which drug would you preferentially choose within a DAPT, and in what percentage?
a. Prasugrel ❍ 25% ❍ 50% ❍ 75% ❍ 100%
b. Clopidogrel ❍ 25% ❍ 50% ❍ 75% ❍ 100%
c. Ticagrelor ❍ 25% ❍ 50% ❍ 75% ❍ 100%

21. At the time of ACS, does the decision to prolong therapy beyond 12 months affect the choice of P2Y12 inhibitor?
a. ❍ Yes
b. ❍ No

22. For you, is the choice of whether to continue DAPT beyond 12 months affected by the age of the patient?
a. ❍ Yes
b. ❍ No

c. ❍ Only in part

23. Which treatment would you choose for an ACS patient older than 75 years?
[Please order the answer options by importance indicating with 1 the preferred or most frequent choice and with 3 the least frequent choice]
...... Prasugrel
...... Clopidogrel
...... Ticagrelor

24. Which treatment would you choose for an ACS patient weighing less than 60 kg?
[Please order the answer options by importance indicating with 1 the preferred or most frequent choice and with 3 the least frequent choice]
...... Prasugrel
...... Clopidogrel
...... Ticagrelor

25. For a patient with CCS, in order to decide how to set the antithrombotic therapy, it is to be evaluated more:
[Please select the most relevant parameter]
a. ❍ The degree of ischemic risk
b. ❍ The degree of hemorrhagic risk
c. ❍ No degree of risk is assessed

26. How long do you routinely treat a patient with CCS post-MI with DAPT?
a. ❍ Maximum for 1 year after the acute event
b. ❍ At least for two to three years after the acute event
c. ❍ Even beyond the third year after an acute event

27. At the 1-year post-MI visit, what element do you consider most important for prolonging DAPT beyond 12 months?
[Please indicate the importance you place on each response option by selecting a score between 1 (least importance/least consideration) and 9 (highest importance/ highest consideration)]

a. That the patient is already being treated with aspirin and ticagrelor	1 2 3 4 5 6 7 8 9
b. That the patient has this indication from the hospital discharge letter	1 2 3 4 5 6 7 8 9
c. That the patient has a favorable ischemic and hemorrhagic risk profile	1 2 3 4 5 6 7 8 9
d. That the patient has tolerated DAPT without adverse events in the first year	1 2 3 4 5 6 7 8 9

28. What is the ideal profile of the candidate patient for prolonged DAPT?
[Express your level of agreement with this statement by selecting a score between 1 (maximum disagreement) and 9 (maximum agreement)]

a. Patient with post-MI CCS, without high hemorrhagic risk, multivessel	1 2 3 4 5 6 7 8 9
b. Patient with post-MI CCS, without high hemorrhagic risk, with high residual ischemic risk due to clinical characteristics (diabetes mellitus, chronic renal failure, acute recurrent events, multidistrict atherosclerosis)	1 2 3 4 5 6 7 8 9
c. Patient with post-MI CCS, without high hemorrhagic risk, at high residual ischemic risk due to procedural features of percutaneous revascularization (common trunk treatment, of three lesions, implantation of three or more stents, bifurcation treatment with two stents, treatment of chronic occlusions or venous grafts,	

total stent length > 60 mm) 1 2 3 4 5 6 7 8 9

29. What is the patient profile that would most benefit from a prolonged DAPT strategy?
[Express your level of agreement for each response option by selecting a score between 1 (maximum disagreement) and 9 (maximum agreement)]

a. Post-MI patient who has tolerated DAPT for 12 months with high residual ischemic risk regardless of type of treatment (PCI versus conservative) 1 2 3 4 5 6 7 8 9
b. Post-MI patient, undergoing PCI, who has tolerated DAPT for 12 months at high residual ischemic risk clinical and/or procedural 1 2 3 4 5 6 7 8 9
c. Post-MI patient, who has tolerated DAPT for 12 months and who has not discontinued it for more than one year, at high residual ischemic risk regardless of the type of treatment (PCI versus conservative) 1 2 3 4 5 6 7 8 9
d. Post-MI patient, who has undergone PCI, who has tolerated DAPT for 12 months and who is at high risk for residual ischemic disease. DAPT for 12 months and who has not discontinued it for more than one year, at high residual ischemic risk clinical and/or procedural 1 2 3 4 5 6 7 8 9

30. What most hinders the use of prolonged DAPT and/or leads to its discontinuation in the patient with post-MI CCS even if potentially useful?
[Express your level of agreement for each response option by selecting a score between 1 (maximum disagreement) and 9 (maximum agreement)]

a. Little time allocated to the visit 1 2 3 4 5 6 7 8 9
b. Low willingness/predisposition of the patient to pursue such therapy 1 2 3 4 5 6 7 8 9
c. Occurrence of side effects 1 2 3 4 5 6 7 8 9
d. Different clinical approaches if the patient is referred to different clinics/physicians 1 2 3 4 5 6 7 8 9
e. Negative influence of caregivers 1 2 3 4 5 6 7 8 9
f. Little interaction between specialist and territory (GP) 1 2 3 4 5 6 7 8 9
g. Little interaction between different specialists (e.g., cardiologist and diabetologist / cardiologist and nephrologist, etc.) 1 2 3 4 5 6 7 8 9
h. Early discontinuation of DAPT 1 2 3 4 5 6 7 8 9

31. What do you value most when deciding to prolong treatment with DAPT in a patient with post-MI CCS?
[Express your level of agreement for each response option by selecting a score between 1 (maximum disagreement) and 9 (maximum agreement)]

a. Residual ischemic risk profile 1 2 3 4 5 6 7 8 9
b. Prevention of new ischemic events 1 2 3 4 5 6 7 8 9
c. Number of vascularizations performed by the patient 1 2 3 4 5 6 7 8 9
d. Fear of bleeding 1 2 3 4 5 6 7 8 9
e. Age of the patient 1 2 3 4 5 6 7 8 9
f. Fragility 1 2 3 4 5 6 7 8 9
g. Comorbidity 1 2 3 4 5 6 7 8 9

32. What do you refer to for the therapeutic management of a patient with post-MI CCS?
[Express your level of agreement for each response option by selecting a score between 1 (highest disagreement/lowest referral) and 9 (highest agreement/most referral)]

a. I follow industry guidelines and consensus documents 1 2 3 4 5 6 7 8 9
b. I use an existing corporate PDTA 1 2 3 4 5 6 7 8 9
c. I use a more empirical approach based on my clinical experience 1 2 3 4 5 6 7 8 9

33. What would you like to see available in your facility to improve care options for patients with CCS?
[Please express your level of agreement for each response option by selecting a score between 1 (maximum disagreement) and 9 (maximum agreement)]
a. Ability to perform remote monitoring 1 2 3 4 5 6 7 8 9
b. Ability to conduct such visits on dedicated agendas and coordinated by your facility 1 2 3 4 5 6 7 8 9
c. Possibility of releasing the report at a later time after the visit, allowing the doctor to be able to compare with other colleagues 1 2 3 4 5 6 7 8 9
d. Presence of a shared diagnostic and therapeutic pathway (PDTA) 1 2 3 4 5 6 7 8 9
e. Dedicated hospital outpatient clinic 1 2 3 4 5 6 7 8 9
f. Possibility to follow up the patient with the same team as in the acute phase 1 2 3 4 5 6 7 8 9
g. Greater availability of budget to be allocated to such projects and/or acknowledgement for physicians engaged in these activities 1 2 3 4 5 6 7 8 9
h. More IT resources for 360° management of patient care (and/or dialogue with GPs, etc.) 1 2 3 4 5 6 7 8 9
i. Collegial sharing of choices 1 2 3 4 5 6 7 8 9
j. Integrated hospital/territory management 1 2 3 4 5 6 7 8 9

34. How important are non-technical skills in your daily work?
[Please express your level of agreement with each answer option by selecting a score between 1 (least important) and 9 (most important)]
a. Use of digital programs/tools 1 2 3 4 5 6 7 8 9
b. Communication with the patient 1 2 3 4 5 6 7 8 9
c. Teamwork with colleagues 1 2 3 4 5 6 7 8 9
d. Other (specify) 1 2 3 4 5 6 7 8 9

QUESTIONNAIRE 2

1. In an organizational follow-up pathway, to increase the number of patients arriving at prolonged DAPT, it would be better to:
a. ❍ Decide based on your own experience
b. ❍ Refer to a pre-established post-acute pathway.

2. To assess ischemic risk, in a patient who is a candidate for prolonged DAPT, which of the following SCOREs would you preferentially choose?
a. ❍ PRECISE DAPT
b. ❍ DAPT
c. ❍ HAS-BLED
d. ❍ GRACE

3. To assess hemorrhagic risk, in a patient who is a candidate for prolonged DAPT, which of the following SCORE would you preferentially choose?

a. ❍ PRECISE DAPT
b. ❍ DAPT
c. ❍ HAS-BLED
d. ❍ GRACE

4. **Which of the following settings seems to best fit the definition of chronic coronary syndromes?**
a. ❍ Patient with history of ACS and/or chronic angina exclusively with angina symptomatology still present (and/or equivalent)
b. ❍ Patient with history of ACS and/or chronic angina exclusively free of anginal symptoms in the chronic (and/or equivalent)
c. ❍ Patient exclusively with history of previous ACS regardless of present symptoms.

5. **In post-ACS patients, how long after the acute event can one speak of a chronic coronary syndrome?**
a. ❍ Beyond 3 months
b. ❍ Beyond 6 months
c. ❍ Beyond 12 months

6. **For an acute phase ACS patient treated with medical therapy alone, which drug would you preferentially choose within a DAPT, and in what percentage?**

a. Prasugrel	❍ 0%	❍ 25%	❍ 50%	❍ 75%	❍ 100%
b. Clopidogrel	❍ 0%	❍ 25%	❍ 50%	❍ 75%	❍ 100%
c. Ticagrelor	❍ 0%	❍ 25%	❍ 50%	❍ 75%	❍ 100%

7. **In an acute phase ACS patient, is the choice of P2Y12 inhibitor contingent on whether, in your judgment, DAPT should be prolonged beyond 12 months?**
a. ❍ Yes
b. ❍ No

8. **For a CCS patient who has not taken ticagrelor (as a P2Y12 inhibitor) in the first 12 months after the acute event would you still assess whether there is eligibility criteria for prolonged DAPT with ticagrelor 60 mg BID?**
a. ❍ Yes
b. ❍ No

9. **Which patient has a more appropriate profile for prolonged DAPT?**
a. ❍ Patient with anatomic-procedural features of higher complexity
b. ❍ Patient with clinical features of high ischemic risk

10. **Which of the following post-MI patients at high residual ischemic risk, who tolerated DAPT well in the first 12 months after an acute event, would benefit most from a prolonged DAPT strategy?**
[Please indicate only the two options that you consider most important]
a. ❍ High-risk patient regardless of treatment type (PCI versus conservative)
b. ❍ High-risk clinical and/or procedural patient undergoing PCI
c. ❍ High-risk patient regardless of treatment type (PCI versus conservative) who has not discontinued DAPT for more than one year
d. ❍ High-risk clinical and/or procedural patient undergoing PCI who have not suspended DAPT for more than one year

11. In a patient with post-MI CCS who could potentially benefit from prolonged DAPT, what hinders its use or leads to its discontinuation?

[Please indicate only the two options that you consider most important]

a. ❍ Occurrence of side effects
b. ❍ Different clinical approaches if the patient is referred to different clinics/physicians
c. ❍ Little interaction between specialist and territory (GP)
d. ❍ Early termination of DAPT

12. To improve the care of patients with CCS, what would you like to see available in your facility?

[Please indicate only the two options that you consider most important]

a. ❍ Greater availability of budget to allocate to such projects and/or acknowledgement for physicians engaged in such activities
b. ❍ Dedicated hospital outpatient clinic
c. ❍ More IT resources for 360° management of patient care (and/or dialogue with GPs, etc.)
d. ❍ Integrated hospital/territory management
