# Supplementary material for: A Therapeutic Pathway in Patients with Chronic Coronary Syndromes: Proposal for Optimization
Source: J Clin Med. 2022 Apr 8;11(8):2091. doi: 10.3390/jcm11082091 (PMC9028169; doi:10.3390/jcm11082091)
Supplement: Supplementary file 1 [file jcm-11-02091-s001.zip › Supplementary Material 2-Contributors.pdf]

Supplementary Material 2 - List of contributors

Francesco Amico, AO Cannizzaro, Catania; Chiara Angeletti, Ospedale Bolognini di Seriate, Seriate; Gianmarco Arabia, ASST Spedali Civili di Brescia, Brescia; Lucia Barbieri, Ospedale Maggiore Policlinico, Milano; Benedetta Bellandi, AUSL Toscana Centro, Firenze; Luca Bettari, Fondazione Poliambulanza Istituto Ospedaliero Multispecialistico, Brescia; Grazia Brienza, ASL Caserta, Caserta; Natale Daniele Brunetti, Università degli Studi di Foggia, Foggia; Francesca Bux, ASL Bari, Bari; Francesca Calì Quaglia, Ospedale Mauriziano, Torino; Paolo Canova, ASST Papa Giovanni XXIII, Bergamo; Alessandro Carbonaro, AOU Policlinico – Vittorio Emanuele, Catania; Massimo Carnazza, ARNAS Garibaldi, Catania; Giovanni Caroli, ASL Brindisi, Brindisi; Francesco Carrata, ASL Bari, Bari; Carlo Caserta, Ospedale Civico, Palermo; Leonardo Cataneo, Ospedale Teresa Masselli San Severo, Foggia; Mariateresa Cellamare, ASL Bari, Bari; Marco Centola, Azienda Ospedaliera San Paolo, Milano; Alberto Cereda, Centro Medico Santagostino, Milano; Francesca Ceriani, ASST Sette Laghi, Varese; Arturo Cesaro, Università degli Studi della Campania "Luigi Vanvitelli", Napoli; Daniela Chiappetta, AO Annunziata, Cosenza; Fabio Chirillo, AULSS 7 Pedemontana - Ospedale di Bassano del Grappa, Bassano del Grappa (VI); Sebastian Cinconze, Ospedale Santa Croce e Carle, Cuneo; Giorgio Conti, ARNAS Garibaldi, Catania; Alessandra Crocetti, AOU di Terni, Terni; Domenico D'Amario, Policlinico Gemelli, Roma; Ovidio De Filippo, AOU Città della Salute e della Scienza, Torino; Emilia De Luca, ASP Cosenza, Catanzaro; Erminia De Lucia, Azienda Ospedaliera Dei Colli - Monaldi-Cotugno-CTO, Napoli; Catia De Rosa, Ospedale Mauriziano, Torino; Francesco De Stefano, Casa di Cura Villa dei Fiori, Acerra (NA); Veronica Della Tommasina, Centro Medico, Pisa; Massimo Di Giorgio, ASL Caserta, Caserta; Paola Di Giovanni, UOC Cardiologia e UTIC PO Mazara, Trapani; Luigi Di Lorenzo, ASL Caserta, Caserta; Agnese Di Naro, ORAS ospedale Riabilitativo Alta Specializzazione Motta di Livenza, Portogruaro (VE); Giovanni Di Tommaso, Ospedale Piedimonte Matese, Piedimonte Matese (CE); Michael Donahue, Policlinico Casilino, Roma; Vittorio Emanuele, UOC Cardiologia, Cosenza;

Andrea Erriquez, AOU di Ferrara, Ferrara; Augusto Esposito, FTGM Ospedale del cuore "G. Pasquinucci", Massa; Andrea Farro, AORN Monaldi, Napoli; Stefano Ferraro, AUSL di Piacenza, Piacenza; Luca Fileti, Ospedale di Ravenna, Ravenna; Alfonso Franzè, SC Cardiologia, ASL TO3 Ospedale degli Infermi, Torino; Sara Fusco, Ospedale di Vaio Fidenza, Parma; Nicola Gasparetto, Cardiologia Ospedale di Treviso - ULSS 2 Marca Trevigiana, Treviso; Laura Gatto, AO San Giovanni Addolorata, Roma; Valerio Giaimo, UOC Cardiologia e UTIC, Enna; Luigi Gianturco, ASST RHODENSE - Ospedale di Passirana, Rho (MI); Sebastiano Gili, Cardiologico Monzino, Milano; Massimo Giordan, Ospedale Santa Maria della Misericordia, Rovigo; Felice Gragnano, AORN "Sant'Anna e San Sebastiano", Caserta; Elena Gribaudo, Città della Salute e della Scienza, Cuneo; Giacinta Guarini, UO Cardiologia 1 AOUP, Pisa; Elisa Guerri, Ospedale Guastalla, Reggio Emilia; Simona Gulino, AO Policlinico, Catania; Alfonso Ielasi, Unità operativa di Cardiologia e UTIC - Istituto Clinico Sant'Ambrogio, Milano; Egle Incalcaterra, ARNAS Civico, Palermo; Elia Iorio, Ospedale Miulli, Bari; Valerio Lanzilotti, AUSL Bologna, Bologna; Claudio Larosa, ASL BAT - Ospedale "L. Bonomo", Andria; Alberto Lavorgna, UO di UTIC - Cardiologia, Teramo; Giacomo Levantesi, Cardiologia - Unità di terapia intensiva cardiologica (Utic), Vasto (CH); Mauro Li Calzi, Ospedale Santa Maria della Scaletta - AUSL Imola, Imola (BO); Nicola Locuratolo, Ospedale San Paolo, Bari; Marco Loffi, ASST Cremona, Cremona; Fabiana Lucà, UOC Cardiologia "Bianchi-Melacrino-Morelli", Reggio Calabria; Valerio Lucci, ASL 1 Avezzano - Sulmona - L'Aquila, L'Aquila; Maria Teresa Manes, UOC Cardiologia, Cosenza; Girolamo Manno, AOU Policlinico Giaccone, Palermo; Francesca Mantovani, Arcispedale S. Maria Nuova - ASMN, Reggio Emilia; Nicola Marchese, Casa Sollievo della Sofferenza, San Giovanni Rotondo (FG); Lucia Marinucci, Ospedale San Salvatore, Pesaro; Flavio Marullo, Ospedale Pineta Grande, Castel Volturno (CE); Silvia Mauri, ASST Ovest Milanese, Legnano; Silvana Micalef, Ospedale Umberto I, Siracusa; Eligio Miccichè, Humanitas Gavazzeni, Bergamo; Andrea Micheli , UOC Cardiologia Treviglio, Bergamo; Marco Alberto Mojoli, Ospedale di Pordenone, Pordenone; Sergio Monaco, Centro di

Diagnostica e Terapia Cardiovascolare, Siracusa; Cinzia Moncalvo, ASL CN1, Cuneo; Salvatore Montalto, Ospedale di Licata, Agrigento; Rocco Montone, Policlinico Gemelli, Roma; Doralisa Morrone, AOU Pisana, Pisa; Saverio Muscoli, PTV, Roma; Roberto Padalino, Casa di Cura Villa dei Fiori, Acerra (NA); Matteo Pagnesi, H San Raffaele, Milano; Angela Palmiotto, ASL Bari, Bari; Marco Pavani, ASL CN1, Cuneo; Dario Pellegrini, ASST Papa Giovanni XXIII, Bergamo; Mauro Pennacchi, AO San Camillo-Forlanini, Roma; Martino Pepe, ASL Bari, Bari; Sante Pierdomenico, Dipartimento Tecnologie Innovative in Medicina - Università "d'Annunzio", Chieti; Christian Piergentili, Unità Coronarica - Ospedale di Feltre, Feltre (BL); Vito Pipitone, AO Ospedali Riuniti Papardo - Piemonte, Messina; Valentina Pitruzzella, AOU Policlinico Giaccone, Palermo; Tommaso Piva, Ospedale Cardiologico Lancisi, Ancona; Davide Presutti, ASL TO4, Torino; Elisabetta Ricottini, Policlinico Campus Bio Medico, Roma; Stefano Rigattieri, Policlinico Sant'Andrea, Roma; Antonio Saino, Cardiologia Unità Coronarica di Seriate - ASST Bergamo Est, Seriate; Alessandra Sanfilippo, ASP, Ragusa; Marilena Scarabelli, AO Cannizzaro, Catania; Francesco Scardaci, AO Cannizzaro, Catania; Adolfo Sciartilli, UOC di Cardiologia - ASL di Lanciano Vasto Chieti, Chieti; Pietro Scicchitano, ASL Bari - PO F. Perinei - Altamura, Bari; Matteo Serenelli, AOU di Ferrara, Ferrara; Salvatore Severino, Casa di Cura " San Michele", Maddaloni (CE); Monica Sicuranza, Fondazione Pascale, Napoli; Nicola Signore, Policlinico di Bari, Bari; Massimo Siviglia, UOC Cardiologia "Bianchi-Melacrino-Morelli", Reggio Calabria; Carlo Andrea Stazi, Ospedale Belcolle, Viterbo; Rocco Stio, AO San Camillo-Forlanini, Roma; Matteo Tebaldi, Arcispedale Sant'Anna, Ferrara; Davide Tomasello, AO Cannizzaro, Catania; Antonio Trivisonno, UOC Cardiologia Ospedale "A.Cardarelli", Campobasso; Chiara Vecchio, ASST Sette Laghi, Varese; Piergiusto Vitulli, ASL 1 Avezzano - Sulmona - L'Aquila, Pescara; Mery Zucchini, AOU Careggi, Firenze.
