# Supplementary material for: A Therapeutic Pathway in Patients with Chronic Coronary Syndromes: Proposal for Optimization
Source: J Clin Med. 2022 Apr 8;11(8):2091. doi: 10.3390/jcm11082091 (PMC9028169; doi:10.3390/jcm11082091)
Supplement: Supplementary file 1 [file jcm-11-02091-s001.zip › Supplementary Material 3-Appropriateness.pdf]

Supplementary Material 3 - Evaluation of appropriateness

Appropriateness indexes

**Item**	**Median**	**IQR**	**IPRAS**	**Assessment**
15. If you work predominantly in an outpatient setting, how important do you think it is that at discharge you are advised whether or not to continue DAPT beyond 12 months?	8	2	6,85	appropriate
16. If you work mainly in an outpatient clinic, what elements hinder your choice to continue DAPT beyond 12 months?				
16.a. Lack of knowledge about PCI intervention	4	6	3,10	uncertain
16.b. Poor knowledge of the patient's ischemic risk profile	4	5	3,10	uncertain
16.c. Fear of the risk of bleeding	5	4	2,35	uncertain
16.d. Limited margin of benefit	3	4	5,35	inappropriate
16.e. Other (specify in next question)	1	2	7,60	inappropriate
17. How would you define a patient with CCS?				
17. a. A patient with suspected CAD and stable angina and/or dyspnea	6	5	2,87	uncertain
17.b. Symptomatic patient	5	6	2,35	uncertain
17.c. Asymptomatic patient (and/or with stabilized symptomatology)	7	4	4,60	appropriate
17.d. Patient who has had an acute event	6	5	3,10	uncertain
17.e. Patient with elective revascularization for more than one year	7	4	6,10	appropriate
17.f. Patient more than 1 year after IM with no further events	8	3	6,10	appropriate
17.g. Patient with elective revascularization for less than 1 year	6	5	3,85	uncertain
17.h. Patient with microvascular or vasospastic angina	6	5	3,85	uncertain
17.i. Patient with recent episode of heart failure and/or diagnosis of left ventricular dysfunction	3	5	4,60	inappropriate
18. Do you consider CCS to be a dynamic disease?	9	2	7,08	appropriate
19. How important is it for a patient with CCS post-MI and multiple risk factors to have the multidisciplinary collaboration of:				
19.a. Diabetologist	8	2	6,85	appropriate
19.b. Nutritionist/Dietitian	7	3	5,35	appropriate

19.c. Psychologist	6	3	3,10	uncertain
19.d. Physical Therapist	5	3	2,35	uncertain
19.e. Nephrologist	6	4	3,85	uncertain
27. At the 1-year post-MI visit, what element do you most consider to extend DAPT beyond 12 months?				
27.a. That he is already on aspirin and ticagrelor 90 treatment	6	4	4,60	uncertain
27.b. That he has this indication from the hospital discharge letter	4	4	3,85	uncertain
27.c. That he has a favorable ischemic and hemorrhagic risk profile	9	1	7,60	appropriate
27.d. That he tolerated DAPT without adverse events in the first year	9	1	7,60	appropriate
28. The ideal profile of the candidate patient for prolonged DAPT is:				
28.a. a patient with CCS post IM, without high hemorrhagic risk, multivessel	9	1	7,60	appropriate
28.b. a patient with post-MI CCS, without high hemorrhagic risk, at high residual ischemic risk by clinical characteristics (diabetes mellitus, chronic renal failure, recurrent acute events, multivessel atherosclerosis)	9	1	8,35	appropriate
28.c. a patient with post-MI CCS, without high hemorrhagic risk, at high residual ischemic risk by procedural characteristics of percutaneous revascularization (common trunk treatment, of three lesions, implantation of three or more stents, bifurcation treatment with two stents, treatment of chronic occlusions or venous grafts, total stent length > 60 mm)	9	1	8,35	appropriate
29. What is the patient profile that would most benefit from a prolonged DAPT strategy?				
29.a. Post-MI patient who has tolerated DAPT for 12 months at high residual ischemic risk regardless of the type of treatment (PCI versus conservative)	8	2	6,85	appropriate
29.b. Post-MI patient, undergoing PCI, who has tolerated DAPT for 12 months at high clinical and/or procedural residual ischemic risk	9	1	7,60	appropriate
29.c. Post-MI patient, who has tolerated DAPT for 12 months and who has not discontinued it for more than one year, at high residual ischemic risk regardless of the type of treatment (PCI	8	2	6,85	appropriate

versus conservative)				
29.d. Post-MI patient, undergoing PCI, who has tolerated DAPT for 12 months and has not discontinued it for more than one year, at high residual clinical and/or procedural ischemic risk.	9	1	7,60	appropriate
30. What most hinders the use of prolonged DAPT and/or leads to its discontinuation in the patient with CCS post-MI even though it is potentially useful?				
30.a. Little time allocated to the visit	4	4	3,85	uncertain
30.b. Patient's unwillingness/predisposition to continue such therapy	6	3	3,33	uncertain
30.c. Occurrence of side effects	7	3	5,35	appropriate
30.d. Different clinical approaches if the patient is referred to different outpatient clinics/physicians	7	3	5,35	appropriate
30.e. Negative influence of caregivers	4	4	3,85	uncertain
30.f. Poor interaction between specialist and territory (GP)	7	3	4,60	appropriate
30.g. Poor interaction between different specialists (e.g., cardiologist and diabetologist/cardiologist and nephrologist, etc.)	6	4	3,85	uncertain
30.h. Early discontinuation of DAPT	7	3	4,60	appropriate
31. What do you evaluate most when making a decision to prolong DAPT treatment in a patient with post-IM CCS?				
31.a. Residual ischemic risk profile	9	1	7,60	appropriate
31.b. Prevention of new ischemic events	8	1	7,60	appropriate
31.c. Number of vascularizations performed by the patient	8	2	7,60	appropriate
31.d. Fear of bleeding	7	3	5,35	appropriate
31.e. Age of the patient	7	3	4,60	appropriate
31.f. Fragility	8	2	6,85	appropriate
31.g. Comorbidity	8	3	6,10	appropriate
32. What do you refer to for the therapeutic management of a patient with post-IM CCS?				
32.a. I follow industry guidelines and consensus documents	9	1	7,60	appropriate
32.b. I use an existing corporate PDTA	3	5	5,35	inappropriate
32.c. I use a more empirical approach based on my clinical	4	4	4,60	uncertain

experience				
33. What would you like to see available in your facility to improve the possibility of care for patients with CCS?				
33.a. Ability to perform remote monitoring	7	3	5,35	appropriate
33.b. Ability to make such visits on dedicated agendas coordinated by your facility	8	3	6,85	appropriate
33.c. Possibility to release the report at a later time after the visit, allowing the physician to compare with other colleagues	5	5	3,10	uncertain
33.d. Presence of a shared Diagnostic-Therapeutic-Assistance Pathway (PDTA)	7	3	4,60	appropriate
33.e. Dedicated hospital outpatient department	7,5	3	6,10	appropriate
33.f. Possibility to follow up the patient with the same team as in the acute phase	6	3	4,60	uncertain
33.g. Greater availability of budget for such projects and/or recognition for physicians engaged in such activities	8	3	5,35	appropriate
33.h. More IT resources for the 360° management of patient care (and/or dialogue with GPs etc.)	8	3	6,85	appropriate
33.i. Collegial sharing of choices	7	3	3,85	appropriate
33.j. Integrated management of hospital and territory	8	3	6,85	appropriate
34. How important are "non-technical skills" in your daily work?				
34.a. Use of digital programs/tools	8	3	6,10	appropriate
34.b. Communication with the patient	9	1	7,60	appropriate
34.c. Teamwork with colleagues	8	2	6,85	appropriate
34.d. Other (specify next)	1	4	6,85	inappropriate

Abbreviations: IQR, interquartile range; IPRAS, Interpercentile Range Adjusted for Symmetry.
